# Predicting survival of patients treated with antibody–drug conjugates in early-phase clinical trials using AI-quantified 3D body composition on CT scans

**DOI:** 10.3389/fonc.2026.1687383

**Published:** 2026-05-13

**Authors:** Matthieu Delaye, Littisha Lawrance, Younes Belkouchi, Felix Wirth, Pierre Decazes, Alexandre Bône, Kaïssa Ouali, Antoine Hollebecque, Cristina Smolenschi, Rastislav Bahleda, Anas Gazzah, Stéphane Champiat, François-Xavier Danlos, Clémence Hénon, Kristi Beshiri, Madona Sakkal, Aurélien Marabelle, Jean-Marie Michot, Vincent Ribrag, Claudia Parisi, Christophe Massard, Vincent Goldschmidt, Pierre Vera, Santiago Ponce-Aix, Nathalie Lassau, Samy Ammari, Capucine Baldini

**Affiliations:** 1 Département d’Innovation Thérapeutique et Essais Précoces, Gustave Roussy - Paris-Saclay University, Villejuif, France; 2 Biomaps, UMR1281 Institut national de la santé et de la recherche médicale (INSERM), commissariat à l'energie atomique (CEA), Centre National de Recherche Scientifique (CNRS), University of Paris-Saclay, Villejuif, France; 3 Guerbet Research, Villepinte, France; 4 Centre de Vision Numérique, CentraleSupélec, Inria, Université Paris-Saclay, Gif-Sur-Yvette, France; 5 Department of Nuclear Medicine, Henri Becquerel Cancer Center, Rouen, France; 6 Institut national de la santé et de la recherche médicale (INSERM), U1015, CIC1428, Immunotherapy Translationnal Research Lab, Villejuif, France; 7 QuantIF-LITIS (EA[Equipe d’Accueil] 4108), Faculty of Medicine, University of Rouen, Rouen, France; 8 Department of Imaging, Gustave Roussy Cancer Campus, University of Paris-Saclay, Villejuif, France; 9 Laboratory of Immunomonitoring in Oncology, Gustave Roussy, Villejuif, France

**Keywords:** anthropometric parameters, antibody-drug conjugates, artificial intelligence, early-phase clinical trial, patient selection

## Abstract

**Background:**

Body composition has a significant impact on the prognosis of cancer patients. However, little is known about its impact on the efficacy and safety of antibody–drug conjugates (ADCs), despite the need for accurate patient profiling to ensure reliable safety data and early signals of activity.

**Methods:**

All patients treated with ADCs in early-phase clinical trials between March 2015 and March 2023 in our institution were retrospectively included in the analysis. Pre-treatment injected CT scans were acquired for all patients. A deep learning software, Anthropometer3DNet, automatically quantified anthropometric parameters in three dimensions (3D) on the acquired CT scans: skeletal muscle mass (SMM), total adipose tissue (TAT), subcutaneous adipose tissue (SAT), visceral adipose tissue (VAT), and lean body mass (LBM). The effect of these anthropometric parameters on progression-free survival (PFS), overall survival (OS), and time in protocol (TIP) was analyzed.

**Results:**

A total of 136 patients were included. The median age, Eastern Cooperative Oncology Group Performance Status (ECOG PS), albumin, and number of previous lines of treatment were respectively 60.8 years (30 to 85), 1 (0–2), 42 g/L [interquartile range (IQR): 39–44], and 3 (IQR: 0–2). The median PFS and OS were 2.6 and 7.9 months, respectively; 90 (66%) patients had experienced toxicity (of which 46 were grade 3–5). Univariate analyses showed that higher SAT [hazard ratio (HR) = 0.67, p = 0.03] and TAT (HR = 0.60, p = 0.01) were significantly associated with longer PFS [median PFS (mPFS) = 2.76 vs. 2.3 and 2.76 vs. 1.9, respectively]. Higher SAT (HR = 0.66, p = 0.04) and higher VAT (HR = 0.65, p = 0.04) were significantly associated with longer OS [median OS (mOS) = 9.34 vs. 7.43 months and 9.27 vs. 6.08 months, respectively]. Higher TAT was associated with longer TIP in both univariate and multivariate analyses (HR = 0.56, p = 0.006). A Royal Marsden Hospital (RMH) prognostic score of 2 or more was associated with PFS, OS, and TIP in both univariate and multivariate analyses (HR = 1.78, 1.89, and 1.74, respectively). All anthropometric parameters were significantly associated with all-grade toxicity in the univariate analysis but not in the multivariate analysis.

**Conclusions:**

Automatic extraction of body composition parameters using artificial intelligence (AI) may help in anticipating the benefits of ADCs in patients included in early-phase clinical trials. Combining anthropomorphic data with clinical and biological data may lead to more refined patient selection.

## Introduction

Antibody–drug conjugates (ADCs) have revolutionized the treatment of many cancers and hematological diseases (1). Their indications are constantly expanding, and numerous trials are underway investigating new ADCs, new indications, combination strategies, or use in early-stage settings (2). By combining a cytotoxic payload with an antibody directed against a tumor target, ADCs deliver cytotoxicity directly to target cells, with significantly enhanced anti-tumor activity compared to standard chemotherapy. However, their effectiveness varies from patient to patient, and there are currently no clearly established biomarkers. In addition, ADCs provoke high rates of toxicity, including high-grade and life-threatening toxicities, some being specific to certain ADCs. As payloads mainly target either tubulin and the mitotic spindle or DNA, toxicities are particularly hematological and neurological, attacking the bone marrow and causing peripheral neuropathy. The toxicity profile is influenced by factors dependent on the ADC itself, such as the payload family, the linker type, or the dosage (3). Other factors, depending on the host, may also play an important role. However, while the pharmacokinetic parameters of ADCs appear to be closer to those of antibodies, little is known about clinico-biological parameters that could influence both ADCs’ efficacy and toxicity.

The influence of body composition on outcomes of patients treated with chemotherapy or targeted therapy has been well established. The first data published focused on sarcopenia that was manually assessed on 2D CT on L3 slices, which was shown to be associated with the toxicity of chemotherapies and multikinase inhibitors (4). It has been suggested that subcutaneous and visceral fat mass may influence prognosis in several cancer subtypes (5, 6), but this can result in inconsistent outcomes across studies, probably due to the heterogeneity of cancer treatment types and the methodology of body composition assessment (7). Longitudinal body composition assessment may yield additional insight into treatment-related changes and outcomes (8). More recently, the development of deep learning software such as the Anthropometer3DNet has enabled complete, automated three-dimensional (3D) analysis of muscle and fat mass, subdividing it into subcutaneous and visceral fat mass (9). Overall survival (OS) in patients treated with anti-angiogenic therapies could be influenced by some parameters of body composition, such as total adipose tissue (TAT), skeletal muscle mass (SMM), subcutaneous adipose tissue (SAT), and visceral adipose tissue (VAT); in contrast, OS in patients receiving immune checkpoint inhibitors could be influenced by SMM and SAT (10, 11). These results refine the understanding of the mechanisms influencing outcomes with anti-cancer treatments and may pave the way for actual treatments that tailor dosage and/or therapeutic strategy, taking into account body composition. No data are available to date about this topic in patients receiving ADCs. These data could be of particular interest in early-phase clinical trials, where a precise patient profile is essential to help generate reliable safety data and an early signal of activity. The aim of our study was to evaluate the prognostic impact and toxicity predictive value of body composition parameters in patients included in early-phase trials investigating ADCs.

## Patients and methods

### Population

In this single-center retrospective analysis, all consecutive patients treated with ADCs in early-phase clinical trials between March 2015 and March 2023 in our institution were included in the analysis. Patients were excluded if they were <18 years old, had not received any injection of the experimental drug, and had a follow-up < 3 months. All patients provided their informed consent to be enrolled in the clinical trial and for the subsequent use of their trial data. This study was approved and registered by our Institutional Review Board according to guidelines for Good Clinical Practice.

### Body composition

**Figure 1:**
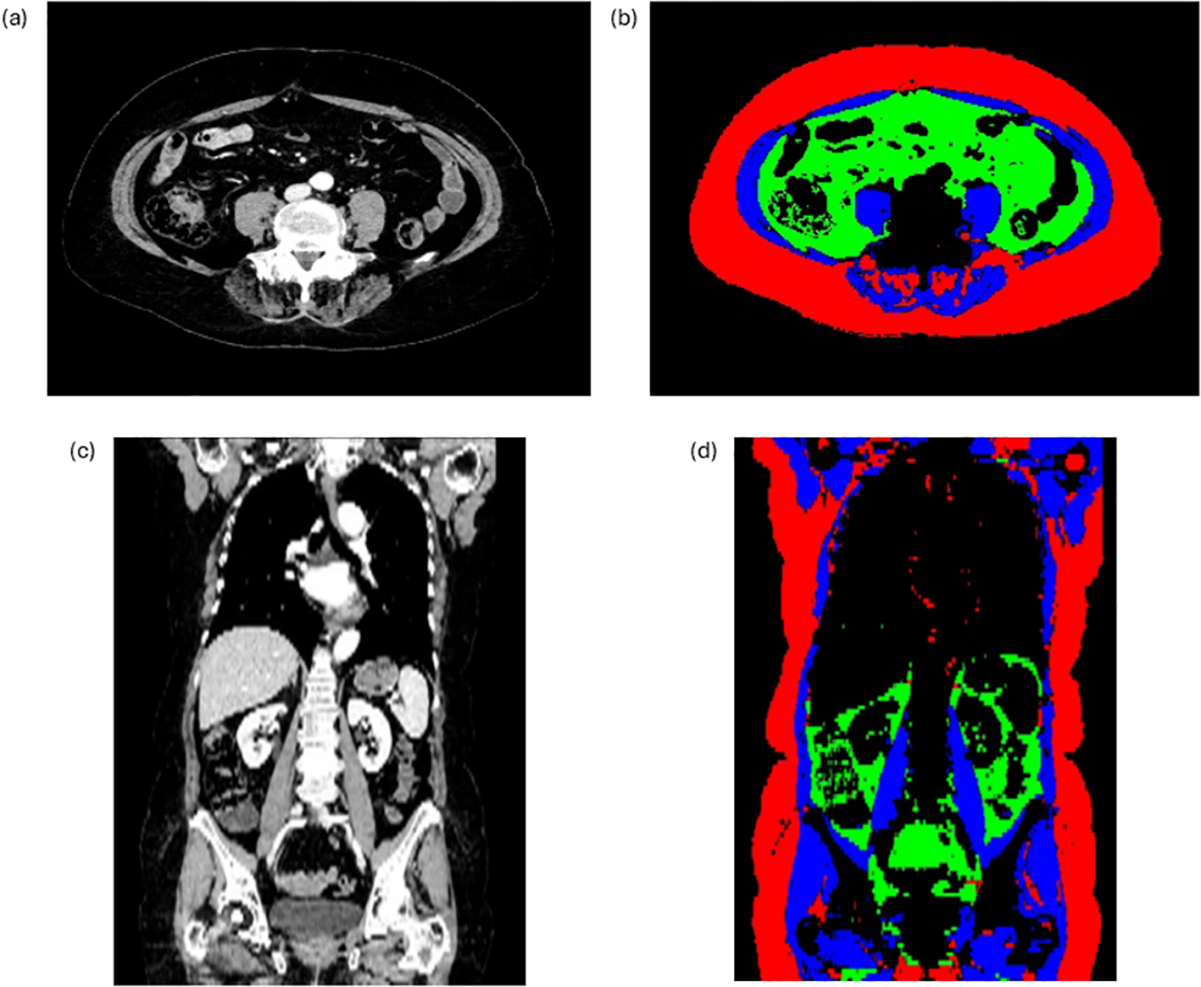
Automatically measured anthropometric parameters in 3D using Anthropometer3DNet. SAT, subcutaneous adipose tissue (red); VAT, visceral adipose tissue (green); TAT, total adipose tissue (green + red); SMM, skeletal muscle mass (blue). Panels a and c depict the unsegmented abdominal axial view and the coronal view, respectively. Panels b and d depict the segmentation of the anthropometric parameters as achieved using Anthropometer3DNet according to the color code described in panels a and c, respectively.

Anthropometric parameters were measured as previously published (11). A deep learning software (Anthropometer3DNet) automatically measured anthropometric parameters in 3D on pre-treatment scans (contrast phase and venous phase), allowing multi-slice measurements of SMM, TAT, VAT, SAT, and lean body mass (LBM) (**Figure 1**). Other clinical and biological parameters were also retrieved based on patients’ clinical files and were evaluated. The scans performed before the treatment had to be acquired approximately 1 month before the treatment started, during the screening period.

### Endpoints

The primary endpoint of the analysis was progression-free survival (PFS), defined as the time from inclusion to progression or death. Other endpoints included OS, defined as the time from inclusion to all-cause death, time in protocol (TIP) (defined as the time elapsed from inclusion to end of treatment, regardless of cause), grade 3–5 toxicity rate, all-grade toxicity rate, and time to high-grade toxicity. Toxicity was graded according to the Common Terminology Criteria for Adverse Events (CTCAE) v4 classification. A grade 3–5 toxicity was considered as high-grade toxicity. Early high-grade toxicity was defined as high-grade toxicity occurring before 90 days of treatment.

Comorbidities were classified according to the Charlson comorbidity index (12). The Royal Marsden Hospital prognostic score (RMH score) was calculated based on albumin level (≥3.5 vs. <3.5 g/dL), lactate dehydrogenase level [less than or equal to the upper limit of normal (≤ULN) vs. >ULN], and the number of metastatic sites (≤2 vs. ≥3 sites) (13). The body mass index (BMI) was classified into four categories based on the values: underweight (BMI < 18.5), normal (18.5 ≤ BMI < 25), overweight (25 ≤ BMI < 30), and obese (BMI > 30).

### Statistical analysis

To check for correlations between the parameters, Pearson’s correlation coefficients were calculated for continuous variables, and the chi-square test was used to check for correlations between categorical variables. The one-way ANOVA was used to check if any continuous variable had a significant impact on a categorical variable. Survival analysis was performed using Cox regression models and the Kaplan–Meier (KM) estimator. Cutoff values were determined using the maximally ranked statistics method. Only the parameters yielding a statistically significant effect in the univariate analyses were considered for the multivariate analyses. Additionally, the effect of the anthropometric parameters on toxicity was also evaluated using a receiver operating characteristic (ROC) analysis and a logistic regression.

## Results

### Population and treatment received

A total of 136 patients were included in the analysis. Their characteristics are listed in **Table 1**. The most frequent tumor types were non-small cell lung cancer (56 patients, 41%) and colorectal cancer (31 patients, 23%). The median age, Eastern Cooperative Oncology Group Performance Status (ECOG PS), albumin, number of previous lines of treatment, and BMI were 60.8 years (30 to 85), 1 (0–2), 42 g/L [interquartile range (IQR): 39–44], 3 (IQR: 0–2), and 24.6 (IQR: 21–27), respectively. Five different ADCs were investigated using three different payloads [deruxtecan, monomethyl auristatin E (MMAE), and DM4]. ADC was studied as monotherapy for 127 patients and in combination with anti-PD1 in nine patients. For all ADCs studied, the dose administered was calculated in milligrams per kilogram of body weight. Patients received a median of four cycles of treatment (1 to 112 cycles), with a median time in study of 85 days (12 to 2,095 days). Overall values and distribution of anthropometric parameters are presented in **Supplementary Table 1**. The correlation of the anthropometric parameters with the clinical parameters and biological parameters, such as the Charlson comorbidity index, ECOG PS, and RMH, was explored. None of the abovementioned parameters showed a significant linear correlation with respect to all four anthropometric parameters. However, BMI was significantly correlated to the anthropometric parameters SAT, VAT, TAT, and SMM with a Spearman’s correlation coefficient of 0.77 (p = 6.2e−28), 0.82 (p = 3.2e−35), 0.82 (p = 2.8e−34), and 0.62 (p = 7.5e−16), respectively.

**Table 1:** Patient characteristics.

Parameter	Overall population (N = 136) N (range)
Age (years), median (min–max)	60.8 (30–85)
Gender, N (%) Male Female	82 (60.3%) 54 (39.7%)
Cancer type, N (%) - Non-small cell lung cancer - Colorectal cancer - Other	56 (41) 31 (23) 49 (36)
ECOG PS, median (min–max)	1 (0–2)
Albumin level (g/L), median (IQR)	42 (39–44)
BMI, median (IQR)	25 (21–27)
RMH prognostic score, median (min–max)	1 (0–2)
Number of previous lines, median (IQR)	3 (2–4)
Type of payload, N (%) - Spindle poisons - Topoisomerase 1 inhibitors	120 (88) 16 (12)
ADC in monotherapy/combination, N (%)	127 (93)/9 (7)
Dose escalation/expansion cohort, N (%)	56 (41)/80 (59)
Median PFS (months) Median OS (months)	2.6 7.9
All-grade toxicity, N (%) Grade 3–5 toxicity	90 (66) 46 (34)

ECOG PS, Eastern Cooperative Oncology Group Performance Status; IQR, interquartile range; BMI, body mass index; RMH, Royal Marsden Hospital prognostic score; ADC, antibody–drug conjugate; PFS, progression-free survival; OS, overall survival.

### Progression-free survival

**Figure 2:**
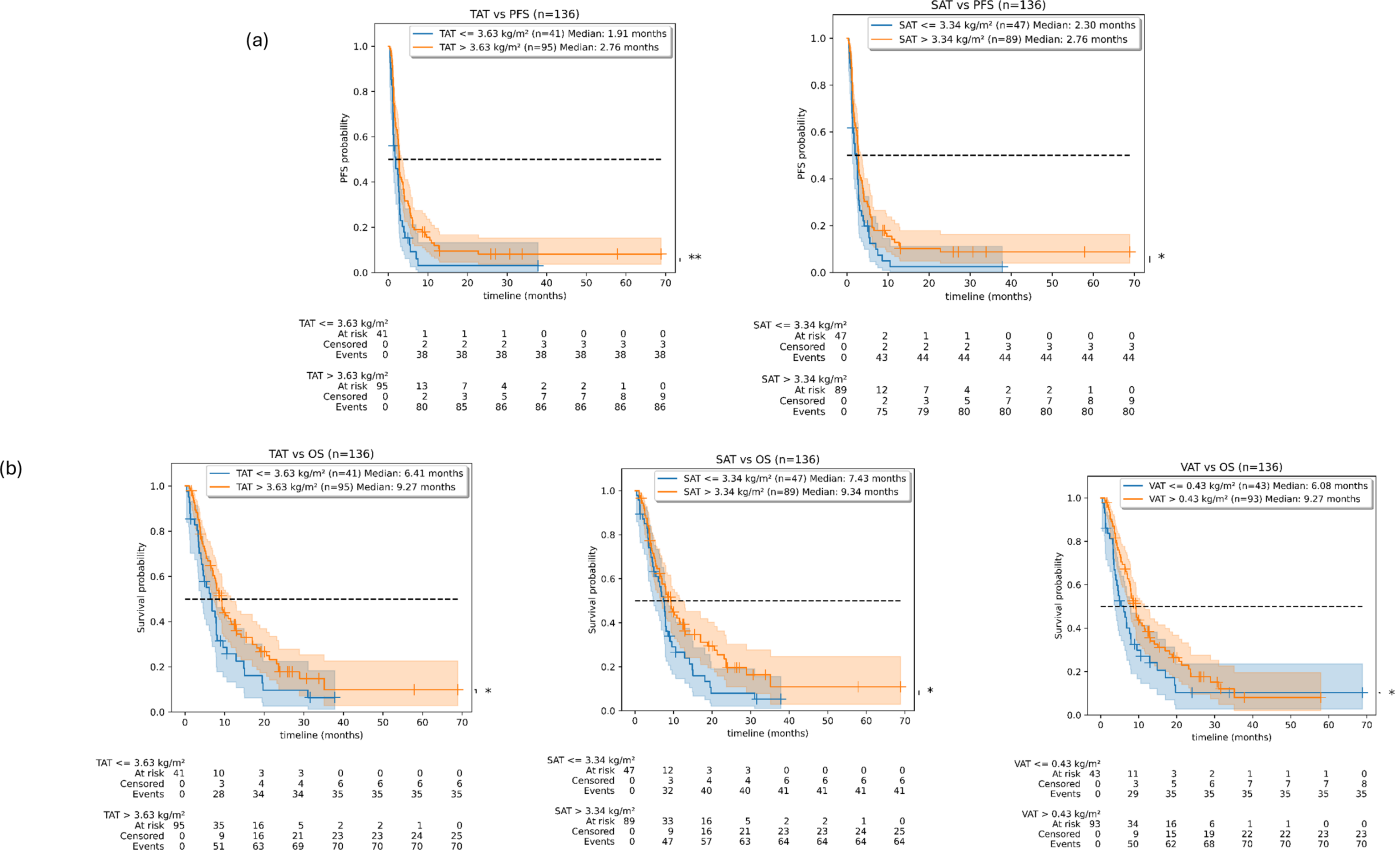
Association between anthropometric parameters and PFS and OS. PFS, progression-free survival; OS, overall survival; TAT, total adipose tissue; SAT, subcutaneous adipose tissue; VAT, visceral adipose tissue. **(a)** Significant difference in PFS between populations with high vs. low TAT (left) and high vs. low SAT (right). **(b)** Significant difference in OS between populations with high vs. low TAT (left), high vs. low SAT (middle), and high vs. low VAT (right).

The median PFS in the overall population was 2.6 months. In univariate analyses, a high RMH prognostic score was significantly associated with a worse PFS [RMH score ≥ 2 or more: 1.38 months vs. 0–1: 2.76 months, hazard ratio (HR) = 1.94, p = 0.009] (**Supplementary Figure 1**). A high number of treatment lines were significantly associated with worse PFS (treatment lines more than 2: 2.43 months vs. 1–2: 3.26 months, HR = 1.8, p = 0.002). The presence of visceral metastases was also associated with a worse PFS (visceral metastases present: 2.56 months vs. absent: 3.75 months, HR = 1.73, p = 0.02). BMI categories were not associated with PFS outcomes. Higher SAT (cutoff: 3.3 kg/m^2^, HR = 0.67, p-value = 0.03) and higher TAT (cutoff: 3.6 kg/m^2^, HR = 0.60, p-value = 0.01) were significantly associated with longer mPFS (2.76 vs. 2.3 months and 2.76 vs. 1.9 months, for SAT and TAT, respectively) (**Figure 2a**). No significant stratification was achieved with SMM (cutoff: 6.1 kg/m^2^), VAT (cutoff: 0.4 kg/m^2^), and LBM (cutoff: 59.2 kg/m^2^). Similarly, the presence of metastatic disease or the number of metastases was not associated with PFS.

Multivariate analyses with high RMH score, high number of treatment lines, the presence of visceral metastases, high SAT, and high TAT as variables identified high RMH score (HR = 1.78, p = 0.03), high number of treatment lines (HR = 1.77, p = 0.004), and the presence of visceral metastases (HR = 1.81, p = 0.02) as independent factors influencing PFS.

### Overall survival

**Figure 3:**
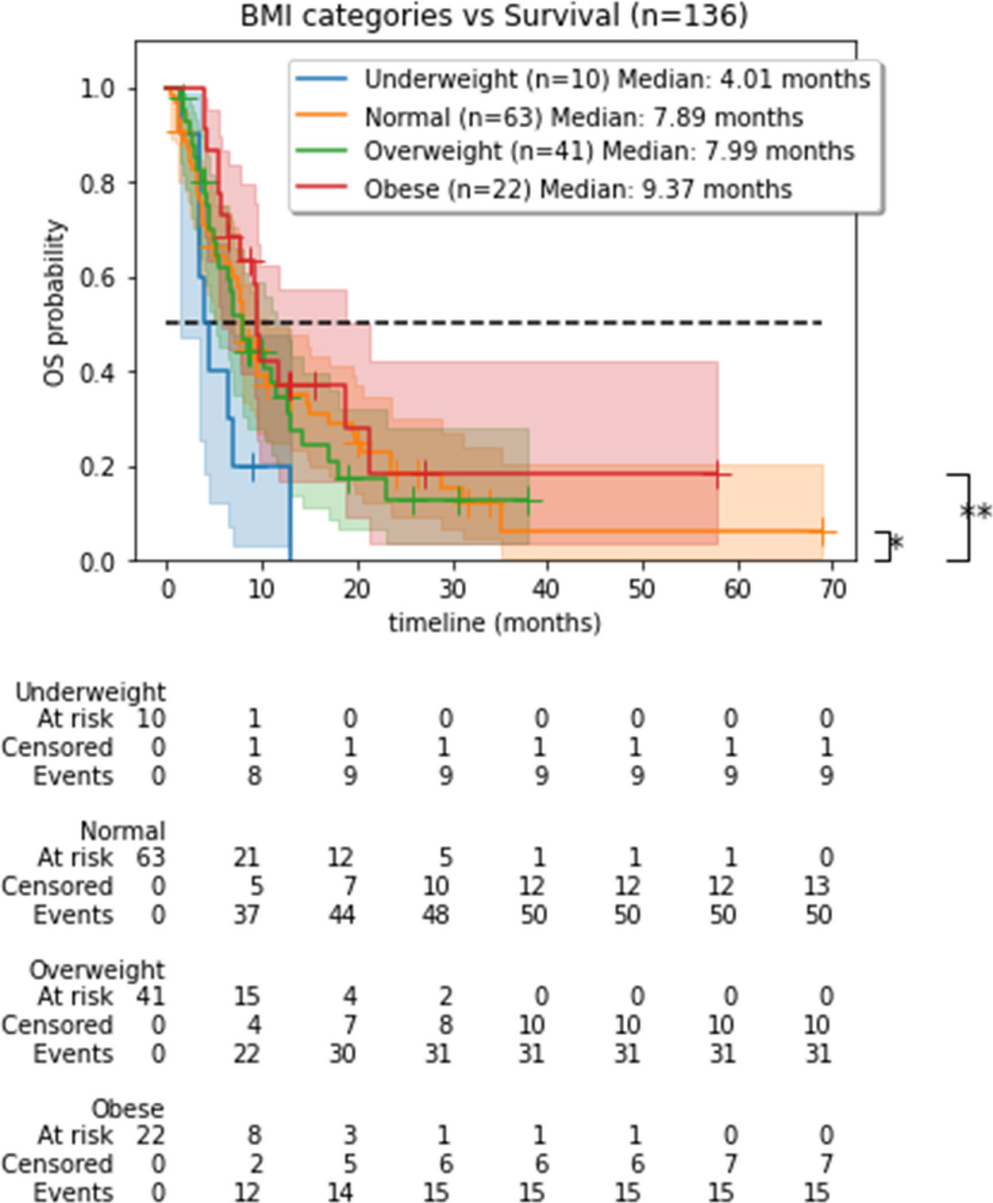
Overall survival by BMI category. BMI, body mass index; OS, overall survival. Kaplan–Meier curves showing a significant difference in OS between body mass index (BMI) categories.

After a median follow-up time of 26.34 months, the median OS in the overall population was 7.9 months. In univariate analyses, a high RMH prognostic score and the number of previous treatment lines were significantly associated with OS [median OS (mOS) = 3.16 months in patients with RMH score of 2 or more versus 8.45 months, HR = 1.86, p = 0.002; mOS = 12.9 months in patients who received two or fewer previous treatment lines versus 7.43 months, and in patients who received more than two previous lines, HR = 1.18, p < 0.005] (**Supplementary Figure 1**). No association was found between OS and the Charlson comorbidity index or other clinico-biological factors except for BMI. A significant separation in the Kaplan–Meier survival curves was observed between the underweight vs. obese (p = 0.01, mOS = 4.01 vs. 9.37 months) and the underweight vs. normal populations (p = 0.04, mOS = 4.01 vs. 7.89 months) (**Figure 3**). The underweight population showed a significant (p = 0.02) HR of 2.23 with respect to OS.

Higher SAT (cutoff: 3.3 kg/m^2^; HR = 0.66, p = 0.04), higher TAT (cutoff 3.6 kg/m^2^; HR = 0.65, p = 0.04), and a VAT higher than or equal to 0.4 kg/m^2^ (HR = 0.65, p = 0.04) were significantly associated with longer OS (mOS = 9.34 vs. 7.43 months, 8.62 vs. 6.41 months, and 9.27 vs. 6.08 months, respectively) (**Figure 2b**) in the univariate analyses. No significant stratification was achieved with SMM and LBM. Similarly, the presence of a metastatic disease, the number of metastases, or the presence of visceral metastases was not associated with OS.

Multivariate analyses with high RMH score, high number of treatment lines, presence in the underweight category, high TAT, high VAT, and high SAT as parameters identified high RMH score (HR = 1.89, p = 0.02) and high number of treatment lines (HR = 2.03, p = 0.001) as independent factors influencing OS.

### Time in protocol

The median TIP was 2.89 months. In univariate analyses, an RMH prognostic score of 2 or more was significantly associated with shorter TIP (1.38 vs. 2.96 months, HR = 2, p = 0.009). Similarly, a patient who received more than two treatment lines (2.53 vs. 4.34 months, HR = 1.88, p = 0.002) spent a significantly shorter TIP (2.53 vs. 4.34 months). The presence of visceral metastases was also associated with shorter TIP (2.76 vs. 3.85 months, HR = 1.8, p = 0.03). The population could not be categorized as having a better or worse TIP based on their BMI categories. Patients with higher TAT (HR = 0.5, p = 0.01) had spent a significantly longer TIP (2.96 vs. 1.91 months). No significant stratification was achieved with SMM, VAT, SAT, or LBM. Similarly, the presence of a metastatic disease or the number of metastases was not associated with TIP.

All multivariate analyses with high RMH score, high number of treatment lines, the presence of visceral metastases, and high TAT as parameters showed high RMH score (HR = 1.74, p = 0.04), high number of treatment lines (HR = 1.9, p = 0.003), the presence of visceral metastases (HR = 1.99, p = 0.02), and high TAT (HR = 0.47, p = 0.0009) as independent factors influencing TIP.

### Toxicity

A total of 90 (66%) patients had experienced all-grade toxicity, of which 46 were high-grade (3–5) toxicities, including one treatment-related death. The median time to high-grade toxicity was 32.5 days (3 to 264 days). The most frequent toxicities experienced were dyspnea (N = 7), hepatic cytolysis (N = 6), and infection (N = 4).

**Figure 4:**
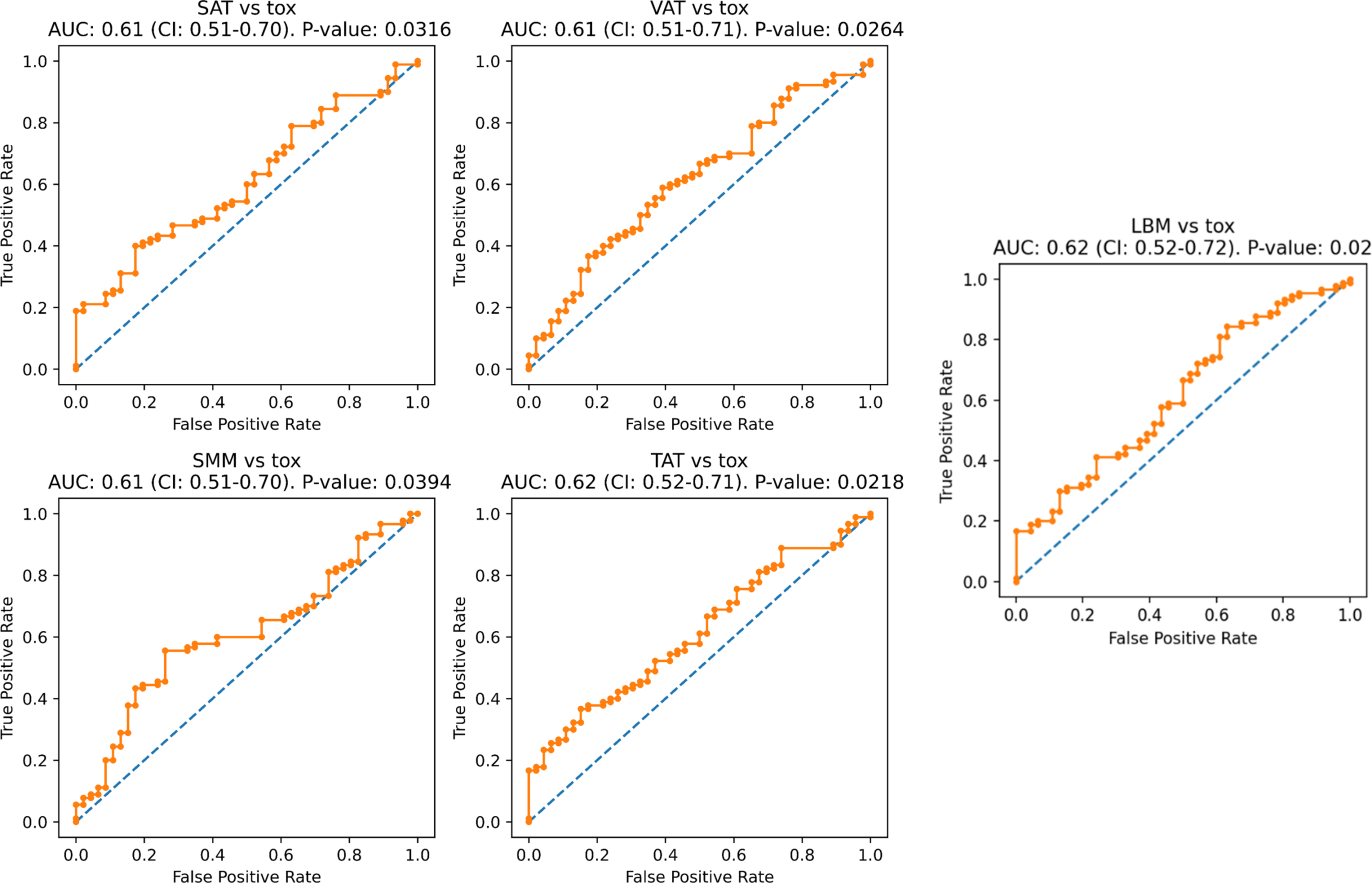
Association between anthropometric parameters and all-grade toxicity. SAT, subcutaneous adipose tissue; tox, all-grade toxicity; AUC, area under curve; VAT, visceral adipose tissue; SMM, skeletal muscle mass; TAT, total adipose tissue. The ROC analysis curves depict the association between anthropometric parameters and all-grade toxicity.

When comparing the correlation between the clinical parameters ECOG PS, BMI, Charlson comorbidity index, and RMH with all-grade toxicity, high-grade (3 and 4) toxicity, and the early high-grade toxicity, no significant correlation was found. When checking for association of toxicity with the four anthropometric parameters, they were significantly associated with all-grade toxicity in the univariate ROC analysis and the logistic regression analysis, but not in the multivariate analysis (**Figure 4**, **Supplementary Table 2**). None of the anthropometric parameters were associated with high-grade toxicity (**Supplementary Table 2**).

## Discussion

This study is the first one to address the question of the impact of body composition on survival and toxicity in patients treated with ADCs. We found that high body fat mass (SAT, VAT, or TAT) and not skeletal muscle mass was associated with PFS and OS in univariate analysis and TIP in multivariate analysis. All anthropometric parameters were associated with all-grade and not high-grade toxicity in univariate analysis. Finally, BMI was only associated with OS and was slightly correlated with all-grade toxicity.

Several hypotheses can be formulated to explain the association between body fat mass and survival.

First, SAT, VAT, and TAT can be interpreted as surrogates of the nutritional status, and higher values correspond to patients with better nutritional status and better intrinsic prognosis. Interestingly, and contrary to what has been observed for anti-angiogenic therapies and immune checkpoint inhibitors (9), no association was found between SMM and survival. This suggests that either SMM is a poorer surrogate of patients’ general condition than body fat mass or that the observed association between body fat mass and survival is explained by something other than a reflection of patients’ intrinsic prognosis.

Second, the amount of fat mass can influence the pharmacokinetics of ADCs. ADCs combine a monoclonal antibody and a payload. Due to their hydrophilic properties, monoclonal antibodies are only distributed in the plasmatic and lymphatic compartments. They are mainly eliminated by catabolism, breaking them down into amino acids, and they are not metabolized by the liver. Payloads are cytotoxic agents that can be topoisomerase 1 inhibitors (deruxtecan and SN38), spindle poisons (MMAE), or DNA-cleaving agents. Spindle poisons (used in 88% of patients in our study) are predominantly lipophilic, whereas topoisomerase 1 inhibitors are predominantly hydrophilic. They are transported in the plasma linked to plasmatic proteins, including albumin. They are mainly metabolized by UGT1A1 and CYP450, with potential impact of genetic polymorphism (UGT1A1) and drug-to-drug interaction (CYP450). Payloads are mainly excreted by the biliary tract and kidneys. We could hypothesize that in the case of higher SAT, the lipophilic payload can be distributed in fatty tissue, accumulate there, and be subsequently released, being therefore responsible for greater efficacy and also greater toxicity. There are no data in the literature to support or refute this hypothesis. Unfortunately, there were no drug or metabolite assays in our study to provide arguments for or against this hypothesis.

Certain clinical parameters exhibit outstanding performance in our study. This is the case of the RMH score, which was associated with PFS, OS, and TIP in our cohort. Our study represents the first demonstration of the validity of this score for ADCs. However, there was no correlation between RMH score and body composition parameters. Thus, body composition parameters could capture additional information that clinical and biological parameters do not. Indeed, CT scanning allows for reliable assessment of muscle and fat mass, whereas albumin (one of the parameters of the RMH score) is influenced by inflammation and liver function. In addition, the location of adipose tissue could also be an important parameter.

BMI categories separated the population with respect to OS with a significant p-value. However, when looking closely at the data, even though the separation in populations was achieved, only the underweight population showed a significant risk ratio, leading to lower survival, thus supporting our results obtained using the anthropometric parameters.

One way forward could be a combined assessment of clinical, biological, and radiological parameters to enable an even more refined evaluation of patients prior to inclusion in early-phase trials studying ADCs and better selection of patients who will benefit from those trials.

Although we used a now well-validated method of body composition assessment, our study was limited by its single-center recruitment, the heterogeneity of the population and drugs, and the absence of drug dosage. Furthermore, the study was carried out in a population of patients enrolled in phase I trials, where the applicability of the results to real-life patients receiving ADC therapy is questionable. However, it should be noted that patients were included at a wide range of stages in their treatment and that the values of body composition parameters were similar to those of previous studies carried out in populations not restricted to early-phase trials (10, 11).

These preliminary results demonstrate that automatically assessing body composition is an easily applicable method that could provide additional data to supplement standard clinical and biological data, enabling patients to be assessed more effectively prior to their inclusion in early-phase trials involving ADCs. However, their usefulness in improving patient selection or even in better tailoring experimental treatment or monitoring needs to be demonstrated in further research. Extending body composition analysis to more homogeneous and larger cohorts of patients could bring new understandings of the behavior of ADCs to optimize their utilization and better select patients who can benefit from these treatments.

## Conclusion

The 3D measurements of high SAT, VAT, and TAT from pre-treatment CT scans were significantly associated with the overall survival of the patient, in addition to the high SAT and high TAT being significantly associated with progression-free survival and the high TAT being associated with TIP. This automatic extraction of body composition parameters using AI may help in anticipating the benefits of ADCs in patients enrolled in early-phase clinical trials and may also be capable of providing insights into the toxicity that will be experienced by the patients during their participation in the trial, which can allow for better recruitment to a phase I clinical trials department. Therefore, combining anthropomorphic data with clinical and biological data may lead to more refined patient selection.

## Data Availability

The raw data supporting the conclusions of this article will be made available by the authors, without undue reservation.

## Supplementary material

Supplementary Figure 1 Association between RMH prognostic score and PFS and OS. RMH, Royal Marsden Hospital prognostic score; PFS, progression-free survival; OS, overall survival. Kaplan Meier curves showing a significant difference in PFS (left) and OS (right) between RMH <=1 vs RMH = 2.

Supplementary Table 1 Overall values and distribution of anthropometric parameters SAT, sub-cutaneous adipose tissue; VAT, visceral adipose tissue; SMM, skeletal muscle mass; LBM, lean body mass; TAT, total adipose tissue. Q1, first quartile. Q3, third quartile.

Supplementary Table 2 Logistic regression of the association between anthropometric parameters and toxicity. SAT, sub-cutaneous adipose tissue; VAT, visceral adipose tissue; SMM, skeletal muscle mass; LBM, lean body mass; TAT, total adipose tissue; CI, Confidence Interval.
