# Supplementary figures and images for: Predicting survival of patients treated with antibody–drug conjugates in early-phase clinical trials using AI-quantified 3D body composition on CT scans
Source: Front Oncol. 2026 May 13;16:1687383. doi: 10.3389/fonc.2026.1687383 (PMC13212237; doi:10.3389/fonc.2026.1687383)

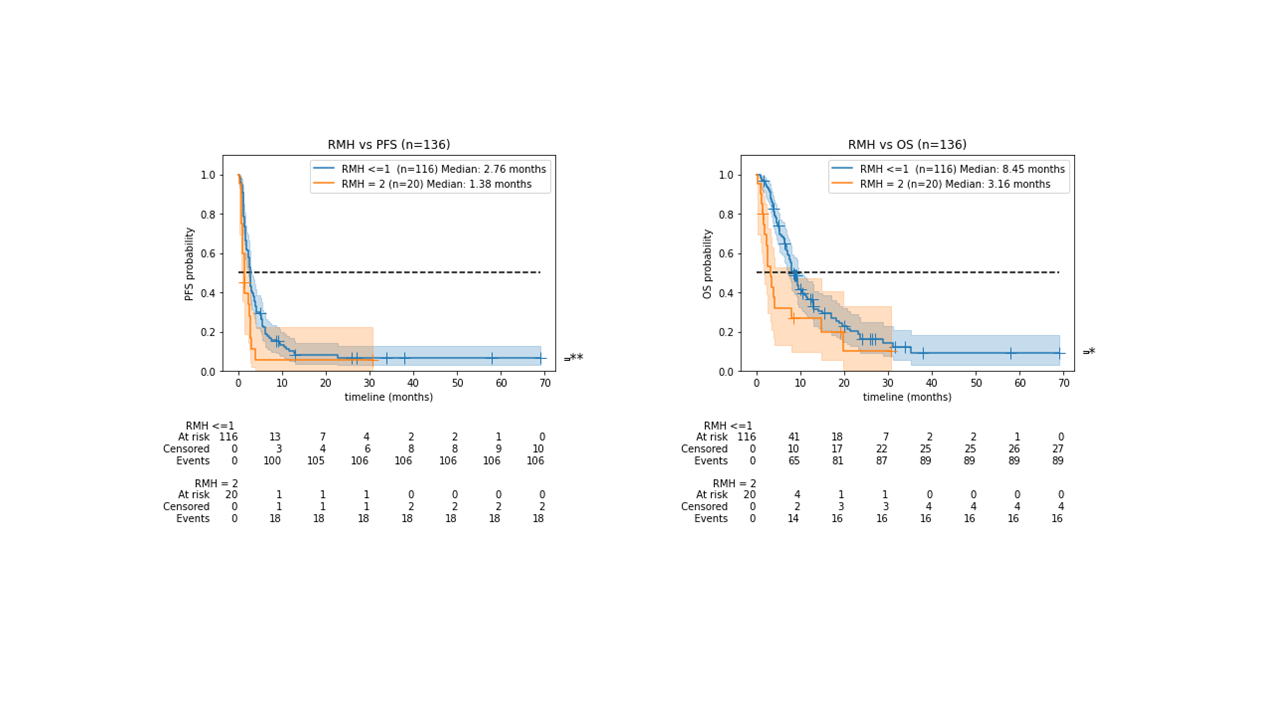

Supplement: Supplementary Figure 1 — Association between RMH prognostic score and PFS and OS. RMH, Royal Marsden Hospital prognostic score; PFS, progression-free survival; OS, overall survival. Kaplan Meier curves showing a significant difference in PFS (left) and OS (right) between RMH <=1 vs RMH = 2. [file Image1.tif]
