# Supplementary material for: Predicting survival of patients treated with antibody–drug conjugates in early-phase clinical trials using AI-quantified 3D body composition on CT scans
Source: Front Oncol. 2026 May 13;16:1687383. doi: 10.3389/fonc.2026.1687383 (PMC13212237; doi:10.3389/fonc.2026.1687383)
Supplement: Supplementary Table 1 — Overall values and distribution of anthropometric parameters SAT, sub-cutaneous adipose tissue; VAT, visceral adipose tissue; SMM, skeletal muscle mass; LBM, lean body mass; TAT, total adipose tissue. Q1, first quartile. Q3, third quartile. [file Table1.docx]

**SAT** (kg/m2)**VAT** (kg/m2)**SMM** (kg/m2)**LBM** (kg/m2)**TAT** (kg/m2)

Median   4.411100                0.789119                 5.719256                66.059011                 5.230958

Q1           2.967617                0.376725                 4.659941                53.898231                 3.422995

Q3           6.287700                1.445532                 6.826488                75.486840                 7.619991

**Supplementary Table 1: Overall values and distribution of anthropometric parameters.**SAT: sub-cutaneous adipose tissue; VAT: visceral adipose tissue; SMM: skeletal muscle mass; LBM: lean body mass; TAT: total adipose tissue. Q1: first quartile. Q3: third quartile.
