# Supplementary material for: Predicting survival of patients treated with antibody–drug conjugates in early-phase clinical trials using AI-quantified 3D body composition on CT scans
Source: Front Oncol. 2026 May 13;16:1687383. doi: 10.3389/fonc.2026.1687383 (PMC13212237; doi:10.3389/fonc.2026.1687383)
Supplement: Supplementary Table 2 — Logistic regression of the association between anthropometric parameters and toxicity. SAT, sub-cutaneous adipose tissue; VAT, visceral adipose tissue; SMM, skeletal muscle mass; LBM, lean body mass; TAT, total adipose tissue; CI, Confidence Interval. [file Table2.docx]

A:

|  | Odds Ratio | 95%CI | p-value |
| --- | --- | --- | --- |
| VAT | 1.93 | 1.08-3.47 | **0.028** |
| SMM | 1.29 | 1.02-1.63 | **0.035** |
| TAT | 1.18 | 1.03-1.34 | **0.015** |
| LBM | 1.03 | 1.01-1.06 | **0.014** |
| SAT | 1.20 | 1.03-1.14 | **0.020** |

B:

|  | Odds Ratio | 95%CI | p-value |
| --- | --- | --- | --- |
| VAT | 1.16 | 0.68-1.96 | 0.587 |
| SMM | 1.01 | 0.85-1.22 | 0.886 |
| TAT | 1.09 | 0.98-1.20 | 0.103 |
| LBM | 1.01 | 0.98-1.03 | 0.525 |
| SAT | 1.11 | 0.99-1.12 | 0.085 |

**Supplementary Table 2: Logistic regression of the association between anthropometric parameters and toxicity.**SAT: sub-cutaneous adipose tissue; VAT: visceral adipose tissue; SMM: skeletal muscle mass; LBM: lean body mass; TAT: total adipose tissue; CI: Confidence Interval
